# Urinary metabolomics-based machine learning for diagnosis of early gastric neoplasia: a retrospective diagnostic case-control study

**DOI:** 10.3389/fonc.2026.1896373

**Published:** 2026-08-04

**Authors:** Mengchen Luo, Feng Chen, Tao Ling, Ting Jin, Maodong Guo, Yifan Lu, Yanlin Hu, Jin Ding

**Affiliations:** 1 Department of Gastroenterology, Affiliated Jinhua Hospital, Zhejiang University School of Medicine, Jinhua, China; 2 Department of Gastroenterology, Zhoushan Putuo District People’s Hospital, Zhoushan, China

**Keywords:** 1-methylnicotinamide, gastric cancer, machine learning, metabolomics, predictive learning models

## Abstract

**Objectives:**

Early gastric cancer (EGC) is often asymptomatic. Non-invasive and scalable screening tools remain limited. We aimed to develop and validate a urinary metabolomics–based machine-learning model for detecting early gastric neoplasia (EGN), including high-grade intraepithelial neoplasia (HGIN) and EGC, and to benchmark it against routine serum markers.

**Methods:**

This was a retrospective diagnostic case-control study using archived urine specimens collected during routine clinical care, with internal split-sample validation and an independent temporal validation cohort. Morning urine samples from two cohorts were profiled by liquid chromatography–mass spectrometry (LC-MS). Cohort 1 was split into a training set and a testing set. LASSO regression was used to identify candidate features, and a random-forest classifier was developed. An external targeted-quantification cohort was used for validation and single-biomarker assessment.

**Results:**

In this retrospective diagnostic case-control study, urinary metabolomics combined with machine learning was used to establish a three-metabolite diagnostic model (3-DM). In the internal testing set, the 3-DM showed discriminative potential with an AUROC of 0.81 (95% CI: 0.600–0.963). In the independent validation cohort, only 1-methylnicotinamide (1-MNA) remained significantly different between NC and EGN, whereas 3-pyridylacetic acid (3-PAA) and phenylacetylglutamine (PAGln) did not show consistent inter-cohort performance. 1-MNA showed a gradual decrease with disease progression, suggesting that it may represent the most reproducible candidate biomarker in this study.

**Conclusions:**

These preliminary findings suggest that urinary metabolomic profiling may have potential for non-invasive detection of EGN. Among the candidate metabolites, 1-MNA showed the most reproducible cross-cohort signal, but its diagnostic performance and clinical applicability require further validation in larger prospective cohorts.

## Introduction

Gastric cancer (GC) remains one of the leading causes of cancer-related deaths worldwide. Although incidence has declined in some regions, the global burden of both diagnosis and mortality remains high, with significant regional disparities. In 2022 alone, there were an estimated 968,350 new cases and 659,853 deaths from GC worldwide—accounting for roughly 7 % of all cancer cases and 9 % of cancer deaths, making GC the fifth most common cancer and the third deadliest globally (1). Clinical evidence clearly shows that early diagnosis and prompt treatment, such as endoscopic mucosal resection or submucosal dissection, can lead to long-term survival rates similar to those of radical gastrectomy, with five-year survival rates often exceeding 90-95%. However, traditional endoscopic surveillance faces limitations due to its invasive nature, dependence on operator skill, and high costs, which restrict access and lower participation in population-based screening efforts (2). This underscores an urgent scientific and public health challenge of developing sensitive, scalable, and non-invasive methods for the early detection of GC.

Metabolomics, which provides a comprehensive analysis of disease-associated metabolic changes, is widely regarded as a direct biochemical indicator of the functional phenotype of neoplasia (3–6). It offers important advantages for biomarker discovery and translational research. Urine-based metabolomics has unique practical advantages over blood-based analyses, owing to its non-invasive collection, greater stability, ease of repeated sampling, and suitability for large-scale population screening (7–9).

The combination of machine learning with high-throughput mass spectrometry has further improved the ability to identify and refine complex metabolic signatures in oncology. In gastric cancer, large-cohort and multi-center metabolomic studies, along with predictive modeling, have shown improved diagnostic accuracy and clinical interpretability compared to traditional protein tumor markers, thus highlighting the synergistic potential of combining metabolomics with machine learning approaches (10–13).

In this study, we used urinary metabolomics combined with machine learning algorithms to explore metabolic reprogramming in the early stages of gastric cancer. The specific goals are: (1) to identify changes in urinary metabolites associated with early disease progression; (2) to find and validate potential biomarkers and parsimonious diagnostic models that remain consistently reproducible across different groups; and (3) to compare these metabolomic models with routine serum tumor markers to examine their translational potential and clinical significance. This study aims to establish reliable urinary metabolic signatures and develop clear diagnostic models, therefore providing a foundation for the development of non-invasive, population-based methods for the early detection of gastric neoplastic lesions.

## Materials and methods

### Study population and sample collection

**Figure 1:**
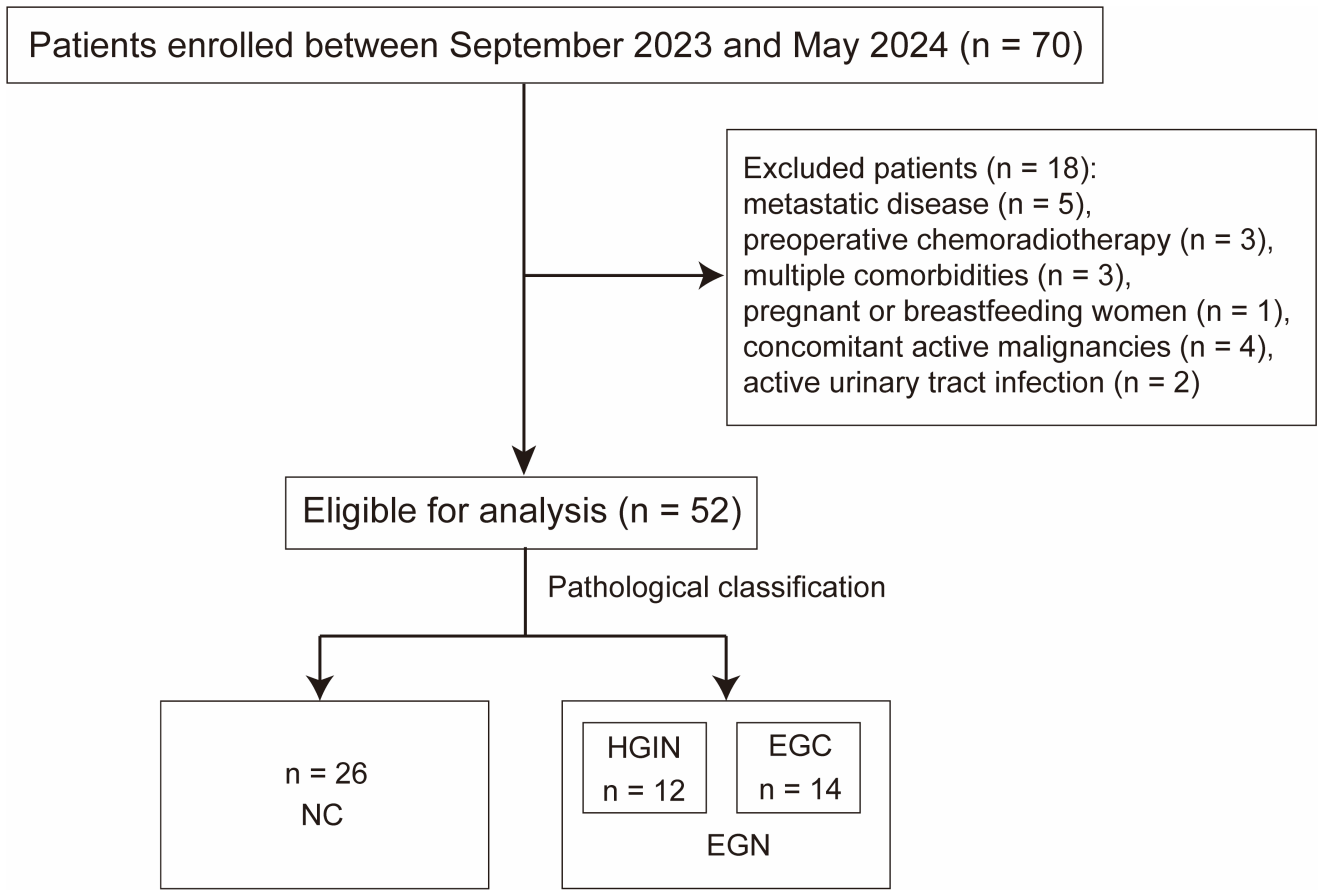
Flow diagram of patient selection and study group classification. A total of 70 patients were initially enrolled between September 2023 and May 2024. After applying exclusion criteria, 52 patients were included in the final analysis. Participants were classified based on pathological findings into NC (n = 26) and EGN (n = 26) groups. The EGN group consisted of HGIN (n = 12) and EGC (n = 14).

This was a retrospective diagnostic case-control study based on archived urine specimens collected during routine clinical care. A total of 52 patients with suspected early GC were recruited between September 2023 and May 2024 from the Department of Gastroenterology at Jinhua Central Hospital. Baseline clinical variables potentially related to urinary metabolite levels, including smoking status, alcohol consumption, body mass index, blood glucose, lipid profiles, liver function, renal function indices, and uric acid, were collected from medical records and compared between groups. Participants with known non-gastric malignancies or severe systemic diseases were excluded according to the predefined exclusion criteria. However, detailed dietary niacin intake, over-the-counter nicotinamide supplement use, and complete medication exposure were not systematically quantified. The inclusion and exclusion criteria are shown in **Figure 1**.

All participants underwent diagnostic upper gastrointestinal endoscopy performed by experienced endoscopists before treatment. During endoscopy, suspicious gastric lesions were carefully inspected using white-light endoscopy, and image-enhanced endoscopy was used when necessary to better delineate lesion margins and surface or vascular patterns. Targeted biopsy specimens were obtained from endoscopically suspicious lesions, including mucosal discoloration, erosion, depression, elevation, irregular surface pattern, or abnormal vascular architecture. For participants classified as normal controls, biopsy specimens were obtained from mucosa without endoscopically visible neoplastic lesions, and the absence of neoplasia was confirmed histopathologically (14–16).

Biopsy specimens were immediately fixed in 10% neutral-buffered formalin, embedded in paraffin, sectioned, and stained with hematoxylin and eosin. Two board-certified GI pathologists, blinded to the metabolomic data and model outputs, independently reviewed all biopsy specimens. Histopathological diagnoses were made according to the WHO classification of tumors of the digestive system (17). In this study, early gastric cancer (EGC) was defined as histologically confirmed gastric adenocarcinoma with invasion confined to the mucosa or submucosa, irrespective of lymph-node status, corresponding to T1 disease in the TNM staging system (15). High-grade intraepithelial neoplasia (HGIN), also referred to as high-grade dysplasia in some classification systems, was defined as a high-grade non-invasive epithelial neoplastic lesion without histological evidence of stromal invasion. Although HGIN is not classified as invasive carcinoma, it is clinically important because of its substantial risk of synchronous carcinoma, histological upgrading after endoscopic resection, and progression to invasive cancer. Therefore, HGIN and EGC were combined as an early gastric neoplasia group for diagnostic model construction, while subgroup analyses were also performed separately for HGIN and EGC. For participants who underwent endoscopic resection, the final histopathological diagnosis and depth of invasion were confirmed using the resection specimen whenever available (18). All EGC cases included in this study were confined to the mucosa or submucosa. Any diagnostic discrepancy was resolved by joint review and consensus. Participants were divided into three groups: non-cancer (NC), HGIN, and EGC (26, 12, and 14 cases, respectively). For diagnostic model development, HGIN and EGC were combined as the early gastric neoplasia (EGN) group because both entities represent clinically relevant early gastric epithelial neoplastic lesions that generally require endoscopic surveillance or resection.

For temporal external validation, an independent set of archived urine specimens from eligible participants examined between June and December 2024 was retrospectively retrieved using the same classification criteria (NC: 14 cases; EGN: 24 cases, including 8 HGIN and 16 EGC). For all included participants, fasting morning urine had been collected before gastroscopy as part of routine clinical care and stored at −80 °C within two hours of collection until analysis. Samples were stored at −80 °C within two h of collection and thawed before testing. After thawing, samples were centrifuged at 12,000 g for 10 min at 4 °C, then filtered to remove particles and proteins.

### Ethics approval and consent to participate

This study was conducted in accordance with the principles of the Declaration of Helsinki. The protocol was reviewed and approved by the Ethics Committee of Jinhua Central Hospital [approval No. (20251110101), date (2025-05-08)].

Urine specimens had been collected during routine clinical care in the Department of Gastroenterology and stored frozen by the Department of Laboratory Medicine. The committee granted a waiver of written informed consent because the study involved minimal risk, used retrospectively stored specimens, involved no additional sample collection or patient contact, and included measures to protect privacy and confidentiality. Only adult participants (≥18 years) were included. This was a retrospective study using previously stored residual urine specimens and de-identified clinical data collected during routine clinical care between 2023 and 2024, so the ethics approval granted in May 2025 represented retrospective waiver approval for the use of archived specimens. No additional sample collection, patient contact, or intervention was performed after study approval.

### Metabolite extraction and untargeted LC–MS acquisition

For metabolite extraction, 100 μL of each urine sample was mixed with 400 μL of extraction solvent (methanol: acetonitrile, 1:1, v/v) containing deuterated internal standards. The mixture was vortexed for 30 sec, then sonicated for 10 min in a 4 °C water bath, and finally incubated for 1 h at −40 °C to precipitate the proteins. Samples were then centrifuged at 12,000 rpm (RCF = 13,800 × g, R = 8.6 cm) for 15 minutes at 4 °C. The supernatants were transferred into clean glass vials for analysis. Quality control (QC) samples were prepared by pooling equal aliquots from all supernatants.

For non-polar metabolites, the same UHPLC system was used, with a Phenomenex Kinetex C18 column (2.1 mm × 100 mm, 2.6 μm) and connected to an Orbitrap Exploris 120 mass spectrometer. The mobile phases consisted of 0.01% acetic acid in water (A) and a mixture of isopropanol and acetonitrile (1:1, v/v) (B). Injection volume and temperature conditions were the same.

The Orbitrap Exploris 120 mass spectrometer was operated in information-dependent acquisition (IDA) mode, controlled *via* Xcalibur software (Thermo). In this mode, the system continuously monitored full scan spectra to select ions for fragmentation. Electrospray ionization (ESI) parameters included: sheath gas flow of 50 Arb, auxiliary gas flow of 15 Arb, capillary temperature set at 320 °C, full MS resolution of 60,000, MS/MS resolution of 15,000, stepped collision energy (SNCE) at 20/30/40, and spray voltage of + 3.8 kV (positive mode) or -3.4 kV (negative mode).

### Data preprocessing and metabolite annotation

The raw dataset included 52 experimental samples and 8 QC samples. Raw data files were converted to mzXML format using ProteoWizard software. Metabolite annotation was performed using a custom R package, which referenced the BiotreeDB (version 3.0) database. A total of 87,888 peaks were initially extracted. To reduce systematic errors and improve biological reliability, data preprocessing was conducted as follows: (1) Peak stability filtering. The reproducibility of each individual peak was evaluated in QC samples. Only peaks with a relative standard deviation (RSD), also known as coefficient of variation (CV), of less than 30% across QC samples were retained. (2) Missing value filtering. Peaks with excessive missingness were filtered according to the 50% missing-value rule, retaining peaks with acceptable detection rates within study groups and/or across the overall dataset. (3) Missing value imputation. Remaining missing values were imputed using one-half of the minimum detected value. This approach was chosen because missing values in untargeted LC–MS metabolomics are often related to low-abundance signals near or below the detection threshold, suggesting an MNAR tendency rather than purely MAR missingness. Compared with k-nearest neighbor or random forest imputation, half-minimum imputation provides a conservative low-value replacement and reduces the risk of artificially increasing weak signals before downstream differential analysis and machine-learning-based feature selection. (4) Normalization. Internal standard-based normalization was applied. After processing, 76,756 peaks were retained. Compounds were identified and classified using HMDB and KEGG databases. Metabolomics data are provided in **Supplementary Table 1**. PLS-DA was conducted using SIMCA software (v14.1), identifying 962 level 1 metabolites. These DEMs were further analyzed through enrichment analysis with the R package clusterProfiler (v3.14.3) using KEGG pathways, followed by soft clustering with Mfuzz.

### Machine-learning model development

Data were split into training and internal test sets in a 7:3 ratio. Performance was additionally evaluated in an independent external cohort. The diagnostic modeling framework was adapted from the workflow reported by Chen et al., in which LASSO-based feature selection was followed by random forest classification [Nature Communications, 2024, DOI: 10.1038/s41467-024-46043-y (11)]. Because of the substantially smaller sample size and the exploratory retrospective design, we did not perform extensive cross-validation-based hyperparameter optimization. LASSO was used as a feature-reduction step to derive a parsimonious candidate metabolite panel from the training set. The LASSO regularization parameter was set to α = 0.05 during exploratory model development, yielding three candidate metabolites with non-zero coefficients: 1-MNA, 3-PAA, and PAGln. These metabolites were then used to train a random forest classifier. The random forest classifier was implemented using scikit-learn. The model was trained with 1000 trees using bootstrap aggregation and the Gini impurity criterion. The predicted probability of EGN was calculated as the average predicted probability across all trees, and a cutoff value of 0.5 was used for binary classification.

In addition to discrimination, model calibration was explored using the Brier score and a grouped calibration plot in the internal testing set. Because the internal testing set contained only 18 participants, predicted probabilities were divided into three groups of equal size for visualization, and calibration assessment was considered exploratory. As an additional internal validation analysis, repeated stratified 5-fold cross-validation was performed to assess the robustness of the fixed three-metabolite model across different sample partitions. The analysis was repeated 100 times, generating 500 fold-level validation estimates. In each fold, the random forest classifier was trained using the prespecified three-metabolite panel and evaluated in the corresponding held-out fold. The same random forest settings used in the primary model were retained, including 1,000 trees, bootstrap aggregation, and the Gini impurity criterion. AUROC values were calculated for each held-out fold and summarized using the mean, the empirical 2.5th–97.5th percentile range, and the proportion of fold-level AUROC estimates exceeding 0.75.

### Targeted quantification for validation

Quantitative analysis of 1-MNA, 3-PAA, and PAGln was performed using UHPLC-MRM-MS. Urine samples were stored at -80 °C and then thawed on ice, thoroughly mixed, and analyzed. A 100 µL aliquot was mixed with 400 µL of pre-cooled acetonitrile: methanol (1:1, v/v) to induce protein precipitation. The mixture was vortexed, sonicated on ice for 15 min, and incubated at -40 °C for 1 h, centrifuged at 12,000 rpm for 15 min at 4 °C. Supernatants (80 µL) were collected for analysis. Chromatographic separation was performed on an Agilent 1290 UHPLC system using a ZORBAX HILIC Plus column (50 × 2.1 mm, 1.8 µm). The mobile phases consisted of solvent A (0.1% acetic acid in water) and solvent B (acetonitrile), with a gradient elution program at 35 °C and an injection volume of 1 µL. An Agilent 6460 triple quadrupole mass spectrometer with an AJS-ESI source was used in MRM mode. For each analyte, optimized Q1/Q3 ion transitions and collision energies were selected, with one transition for quantification and one to two additional transitions for qualitative confirmation. External standards were used for calibration, and standard curves were generated from serially diluted calibration solutions using least-squares regression with 1/x weighting (R² ≥ 0.997). Analyte concentrations were calculated from instrument responses, adjusted for dilution factors. The method’s performance was validated by spiking recovery (81.3–91.2%) and repeatability tests (RSD < 1.9%). Analytical sensitivity was evaluated based on the signal-to-noise ratio, resulting in lower limits of detection (LLOD) ranging from 0.08 to 4.89 nmol/L and lower limits of quantification (LLOQ) ranging from 0.15 to 9.77 nmol/L. Under these analytical conditions, all 3 metabolites showed consistent retention times and sharp, well-resolved peaks, meeting the criteria for reliable quantification in clinical specimens.

### Statistical analysis

Data were compared between two unpaired groups using the two-sided Wilcoxon rank-sum test, while comparisons across three or more groups were performed with the two-sided Kruskal–Wallis test. A *p*-value < 0.05 was considered statistically significant. Data were statistically analyzed using GraphPad Prism (v10.1.2), R (v4.4.2), SPSS Statistics 27.0 (IBM), and Python (scikit-learn v0.24.1).

## Results

### Patients, data collection, and study design

**Figure 2:**
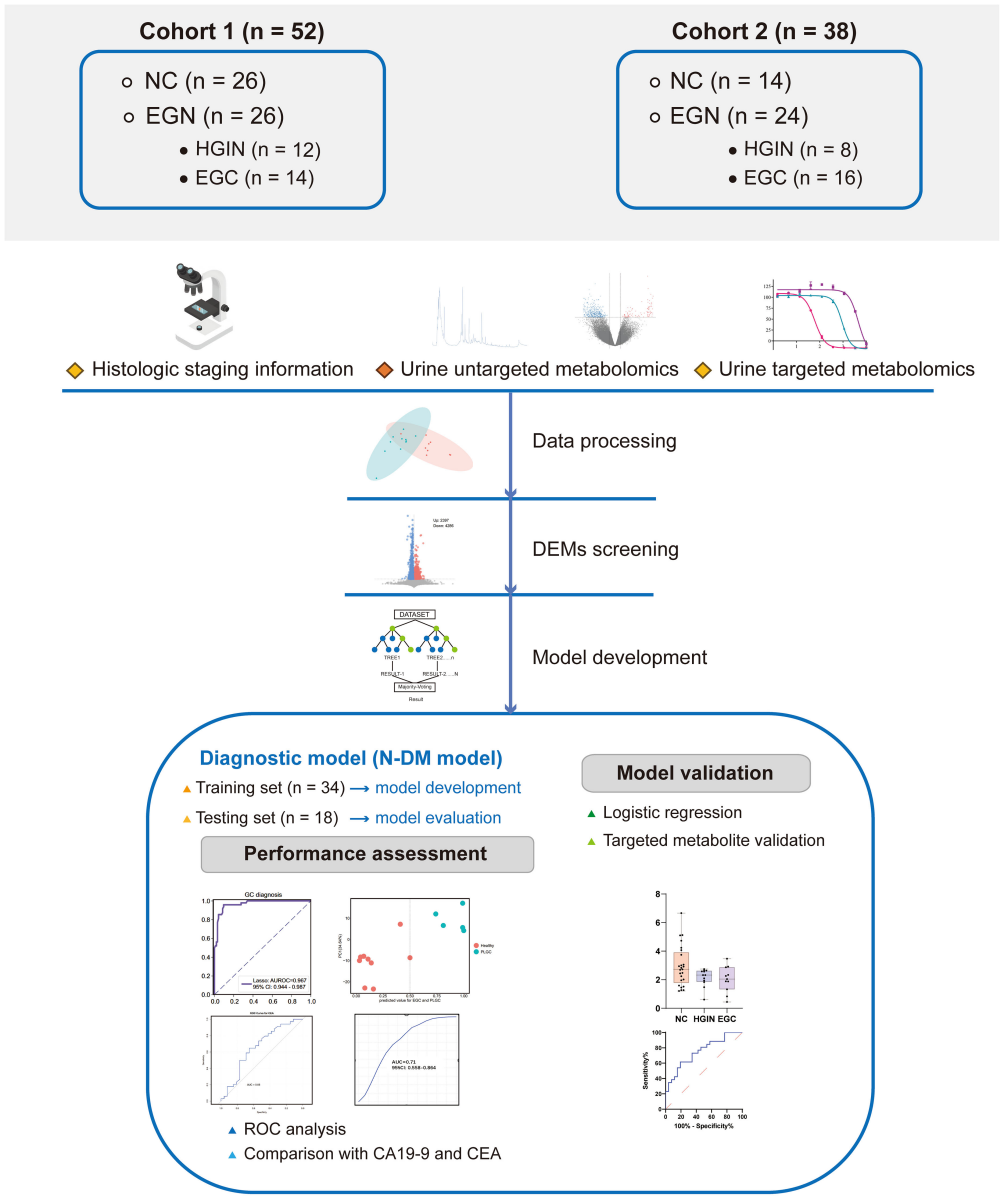
Schematic overview of study design and diagnostic model development. Cohort 1 (n = 52) consisted of NC (n = 26) and EGN (n = 26), including HGIN (n = 12) and EGC (n = 14). Cohort 2 (n = 38) included NC (n = 14) and EGN (n = 24). Urine metabolomics (untargeted and targeted) and histologic staging information were integrated for data processing and differential metabolite screening. A diagnostic model (N-DM model) was developed using a training set (n = 34) and evaluated in an internal testing set (n = 18). Model performance was assessed using ROC analysis and compared with conventional tumor markers (CA19–9 and CEA).

The overall workflow of this study is shown in **Figure 2**. Fasting urine samples were collected from 70 suspected early GC patients. Histopathological results from endoscopic biopsy and subsequent endoscopic resection specimens were used to categorize participants into NC (n = 26) and EGN (n = 26). The EGN group was further divided into HGIN (n = 12) and EGC (n = 14). Clinical characteristics of the study population are summarized in **Table 1**. Baseline characteristics, including smoking status, alcohol consumption, BMI, blood glucose, lipid profiles, liver function, renal function indices, and uric acid, were generally comparable between the NC and EGN groups. For independent temporal validation, 38 additional archived urine specimens were analyzed using targeted metabolite quantification to evaluate the robustness and clinical applicability of the model.

**Table 1:** Characteristics of the study participants.

Characteristics	Group		*p* value
	NC (26)	EGN (26)	
gender (M/F)	16/10	20/6	0.375^a^
Age (y,mean ± SD)	64.1 ± 8.2	69.0 ± 6.8	0.054^b^
BMI (kg/m^2^,mean ± SD)	24.0 ± 4.1	21.9 ± 3.6	0.063^b^
HP infection, n%			0.598^a^
infected	8 (30.8)	8 (30.8)	
uninfected	12 (46.2)	9 (34.6)	
Unknown	6 (23.0)	9 (34.6)	
Smoke, n%			0.059^a^
never Smoke	21 (80.8)	16 (57.7)	
past Smoke	0	5 (19.2)	
Current Smoking	5 (19.2)	5 (23.1)	
Drink, n%			0.532^a^
no Drink	18 (69.2)	20 (76.9)	
Drinking	8 (30.8)	6 (23.1)	
Blood pressure			
Systolic	133.8 ± 14.4	129.9 ± 16.1	0.363^b^
Diastolic	80.1 ± 10.7	78.1 ± 9.9	0.495^b^
Laboratory finding			
random glucose (mmol/L)	7.0 ± 1.4	6.7 ± 1.5	0.409^b^
TC^*^ (mmol/L)	4.8 ± 0.8	4.4 ± 1.1	0.086^b^
TG^**^ (mmol/L)	1.9 ± 1.2	1.3 ± 0.7	0.071^b^
ALT^***^ (U/L)	19.3 ± 11.8	18.1 ± 7.9	0.659^b^
AST^****^ (U/L)	23.9 ± 9.9	24.8 ± 8.6	0.729^b^
ALP^#^ (U/L)	78.8 ± 17.4	70.4 ± 20.6	0.119^b^
TBil^##^ (μmol/L)	13.6 ± 3.6	13.7 ± 5.8	0.898^b^
Total Bile Acid (μmol/L)	5.5 ± 3.6	7.1 ± 8.3	0.366^b^
Scr^###^ (μmol/L)	76.9 ± 15.5	74.3 ± 13.4	0.533^b^
BUN^####^ (mmol/L)	5.7 ± 1.0	5.4 ± 1.0	0.293^b^
UA (μmol/L)	322.6 ± 81.0	323.7 ± 88.0	0.964^b^
Histologic diagnosis, n%			
High-grade intraepithelial neoplasia	/	12 (46.2)	
Early gastric cancer	/	14 (53.8)	

^a^χ^2^ test;

^b^independent-samples T test. TC^*^, total cholesterol; TG^**^, triglycerides; ALT^***^, alanine aminotransferase; AST^****^, aspartate aminotransferase; ALP^#^, alkaline phosphatase; TBil^##^, total bilirubin; Scr^###^, serum creatinine; BUN^####^, blood urea nitrogen; UA, uric acid.

Non-targeted LC-MS-based metabolomic profiling was performed on each urine sample to identify metabolic changes. After data normalization, comparison between the NC and EGN groups identified 39 differentially expressed metabolites (DEMs). Machine learning methods were then used to explore the association between metabolic characteristics and pathological grades. Based on this analysis, a diagnostic model, known as the “ three-metabolite diagnostic model (3-DM) “, was developed using the most effective metabolites. Model performance was evaluated through multiple validation strategies. For an independent validation, 38 additional urine samples (14 NCs, 24 EGNs, including 8 HGINs and 16 EGCs) were analyzed using targeted metabolite quantification to evaluate the robustness and clinical applicability of the model.

### Urinary metabolomics landscape in EGN patients

**Figure 3:**
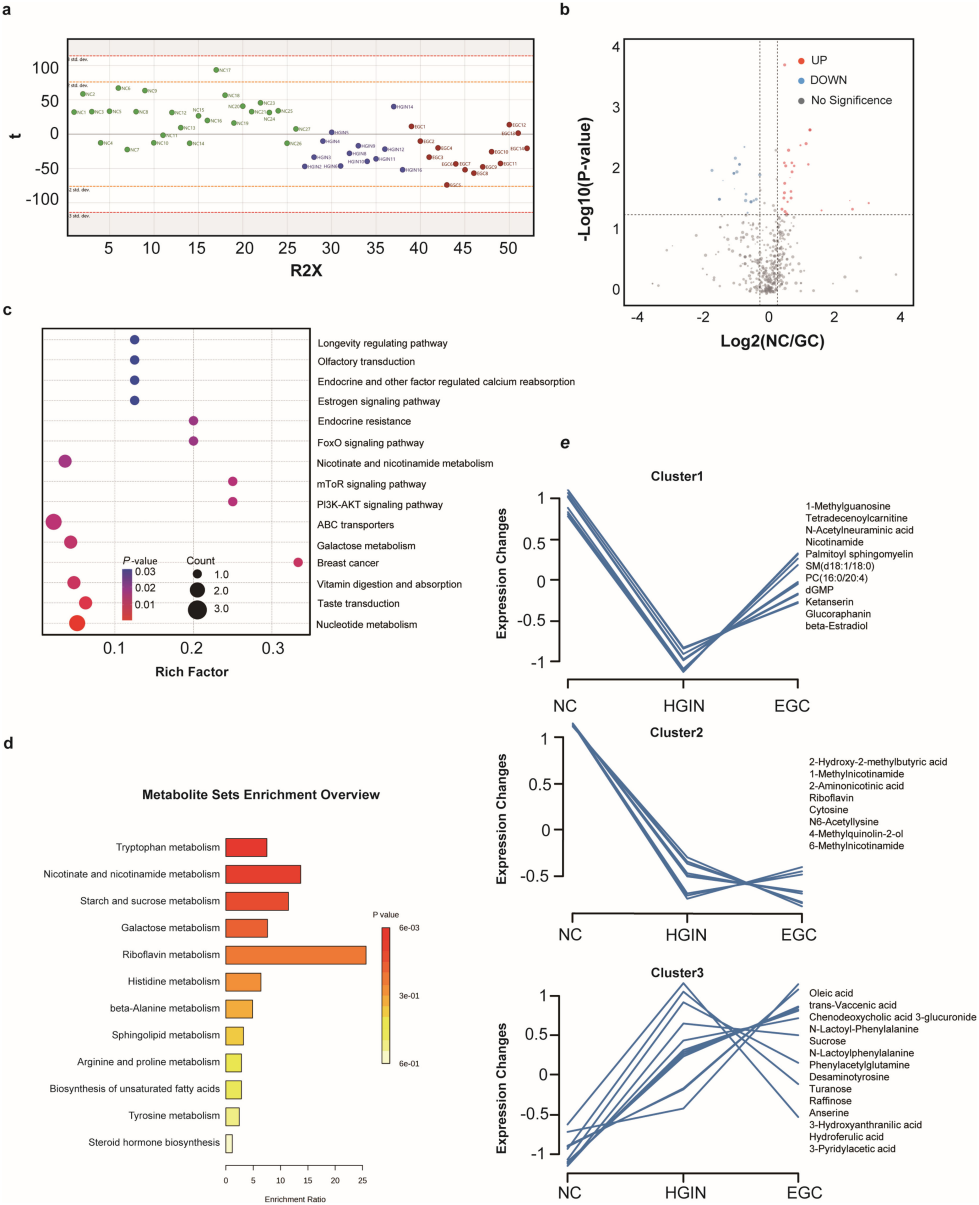
Differential metabolite profiling and enrichment analysis. **(a)** PLS-DA score plot depicting separation between NC and EGN groups. **(b)** Volcano plot showing differentially expressed metabolites, with upregulated metabolites in red and downregulated in blue (two-sided Wilcoxon rank-sum test, Benjamini-Hochberg correction; VIP ≥ 1, FDR < 0.05, fold change > 1.2 or < 0.83). **(c)** Pathway enrichment analysis of significant metabolites, displaying associated *p*-values and enrichment factors. **(d)** Overview of enriched metabolite sets, with key metabolic pathways indicated. **(e)** Mfuzz clustering illustrating metabolic trajectories across NC, HGIN, and EGC groups, with cluster-specific metabolites shown alongside.

To characterize metabolic changes in disease stages, multi-step screening was used to identify DEMs associated with EGN. Partial least squares discriminant analysis (PLS-DA) differentiated EGN from NC specimens (**Figure 3a**), confirming metabolic reprogramming between groups. This supported subsequent DEM identification and pathway analysis.

Compared to the NC group, 39 DEMs showed significant changes in the EGN group (**Figure 3b**). To explore the underlying biological role of these different metabolites and the main pathways involved, enrichment analysis revealed strong associations with cancer-related signaling pathways, such as mTOR, PI3K–Akt, and FoxO pathways (**Figure 3c**). These pathways are crucially involved in regulating tumor growth, survival mechanisms, cellular migration, and therapeutic resistance (19–21). Furthermore, metabolite set enrichment analysis (MSEA) indicated broad metabolic changes, with riboflavin metabolism being the most prominently enriched pathway (**Figure 3d**).

To examine dynamic metabolic changes during disease progression, Mfuzz soft clustering was applied to the 39 DEMs (**Figure 3e**). Three key metabolic trajectories were identified (Clusters 1–3). Cluster 2, which includes 2-hydroxy-2-methylbutyric acid and 1-MNA, showed a continuous decline, whereas metabolites in Cluster 3 demonstrated a steady increase with disease progression.

### Construction of a predictive model for EGN using machine learning

**Figure 4:**
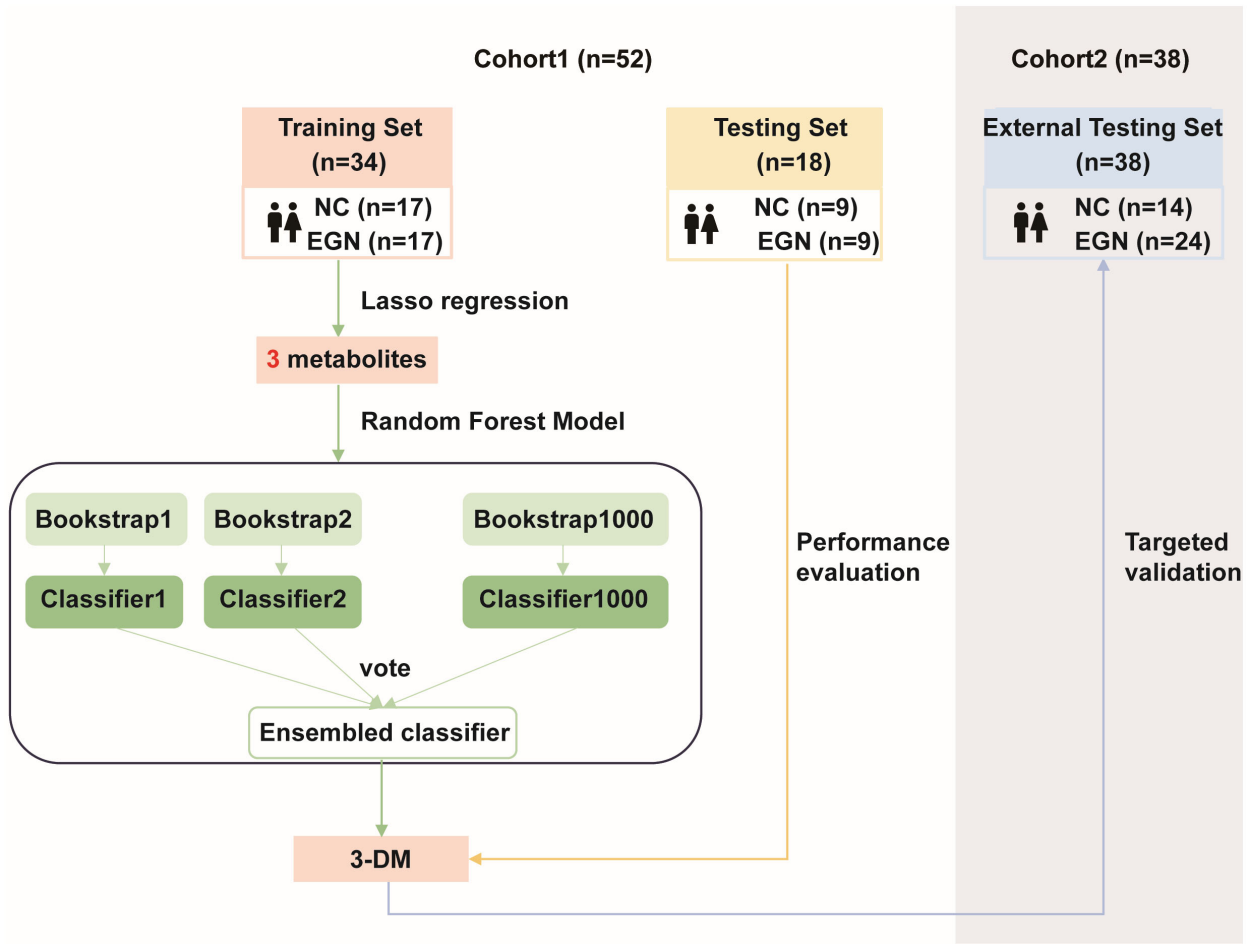
Workflow for development and validation of the 3-DM. Cohort 1 (n = 52) was divided into a training set (n = 34) and a testing set (n = 18). Lasso regression was used on the training set to identify three metabolites, which were then used to construct a random forest model with bootstrapping and ensemble aggregation. Model performance was evaluated in the testing set, and external validation was conducted with Cohort 2 (n = 38) using targeted metabolite analysis.

In this study, a diagnostic model comprising three metabolites was developed to discriminate EGN (**Figure 4**). To maintain strict separation between training and evaluation data, Cohort 1 (n = 52) was randomly divided into a training set (n = 34; NC = 17, EGN = 17) and a testing set (n = 18; NC = 9, EGN = 9).

During the training phase, LASSO regression was used to reduce dimensionality in metabolic features, suppress multicollinearity, minimize overfitting risk, and improve interpretability and clinical relevance. This method identified three discriminative metabolites: 1-MNA, 3-pyridylacetic acid (3-PAA), and phenylacetylglutamine (PAGln).

**Figure 5:**
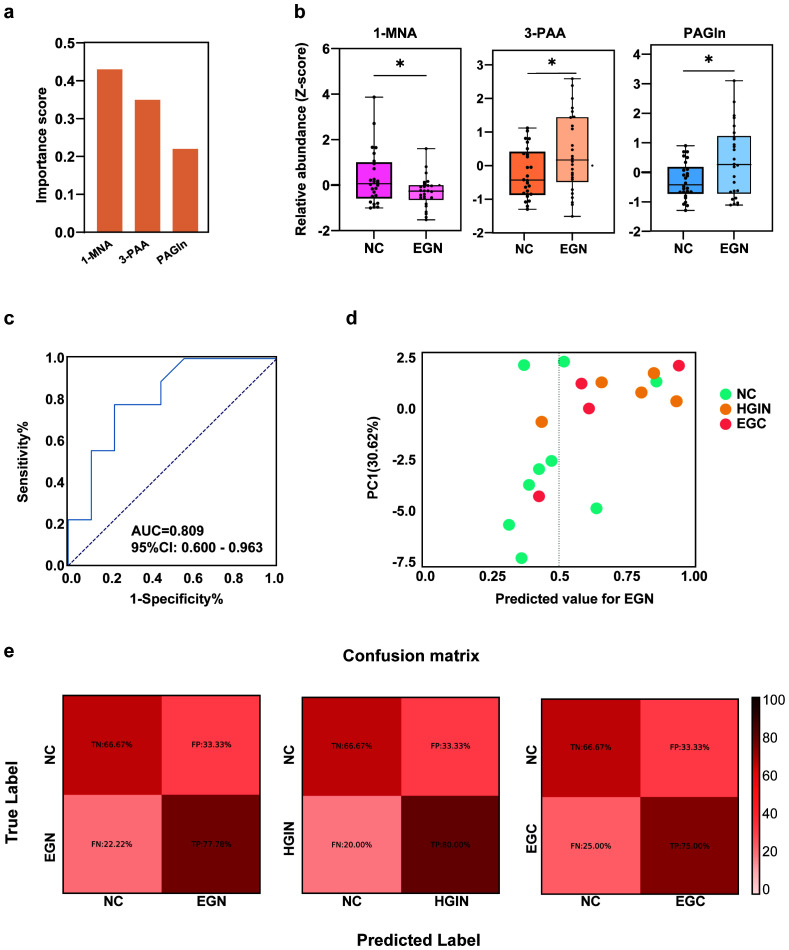
Diagnostic performance of the 3-DM. **(a)** Feature importance scores of the three selected metabolites in the random forest model. **(b)** Box plots comparing relative abundances of 1-MNA, 3-PAA, and PAGln between NC and EGN groups (*p < 0.05, **p < 0.01, ***p < 0.001). **(c)** ROC curve of the 3-DM, showing an AUC of 0.809 (95% CI: 0.600-0.963) with respective sensitivity and specificity. **(d)** Predicted probability distribution among NC, HGIN, and EGC samples. **(e)** Confusion matrices summarizing classification outcomes of the 3-DM for NC, EGN, HGIN, and EGC, with true negatives (TN), false positives (FP), false negatives (FN), and true positives (TP) for each stage.

To capture nonlinear relationships and potential interaction effects, a random forest model was trained using these three metabolites. The model was constructed with 1,000 bootstrap resamples, and ensemble classification was performed through majority voting to decrease variability from individual learners. Feature importance analysis revealed similar contributions from all three metabolites, with 1-MNA having a slightly higher influence (**Figure 5a**). To confirm biological consistency and highlight group-specific differences, the relative abundances of the metabolites were compared between the NC and EGN groups (**Figure 5b**).

In the testing set, the 3-DM achieved an AUC of 0.809 (95% CI: 0.600-0.963) (**Figure 5c**). Model performance was further illustrated by plotting predicted probabilities against actual disease status (NC/EGN). Using a threshold of 0.5, most NC samples were classified below the cutoff, while HGIN and EGC samples were predominantly classified above it. However, there was no clear separation between HGIN and EGC (**Figure 5d**). This finding indicates that the 3-DM primarily captured metabolic differences between non-neoplastic controls and early gastric neoplastic lesions, rather than stage-specific differences between HGIN and EGC. Therefore, the subgroup results should be interpreted as exploratory. To evaluate categorical performance, a confusion matrix was generated, and key metrics were calculated. In binary classification (“Healthy vs. All Stages”), the sensitivity was 77.78%. Stratified analysis revealed sensitivities of 80.00% for HGIN and 75.00% for EGC (**Figure 5e**). In addition to discrimination, model calibration was explored in the internal testing set. The 3-DM yielded a Brier score of 0.194 (**Supplementary Figure 1**). The grouped calibration plot showed approximate agreement between predicted probabilities and observed EGN rates, particularly in the intermediate- and high-probability groups, although the model tended to overestimate risk in the low-probability group. Because the internal testing set contained only 18 participants, this calibration analysis was considered exploratory. To further examine the robustness of the fixed three-metabolite classifier, repeated stratified 5-fold cross-validation was performed with 100 repetitions. Across the resulting 500 held-out fold evaluations, the mean AUROC was 0.680, with an empirical 2.5th–97.5th percentile range of 0.440–0.960. An AUROC greater than 0.75 was observed in 33.2% of the fold-level evaluations. The broad distribution of AUROC estimates indicated that model performance was sensitive to sample partitioning, which was expected given the small number of participants in each validation fold. These findings suggest moderate average discrimination but substantial uncertainty in the internal performance estimate. The distribution of cross-validated AUROC values is shown in **Supplementary Figure 2**. Error analysis on the test and external sets showed that most false positives shared overlapping clinical profiles, whereas false negatives clustered near the decision threshold. Representative cases are summarized in **Supplementary Table 2**. Overall, these findings suggest that the 3-DM may have potential to distinguish NC from clinically relevant early gastric neoplastic lesions, although it should not be interpreted as a model for reliably discriminating HGIN from EGC.

### Comparison of the diagnostic performance of the 3-DM with traditional biomarkers

**Figure 6:**
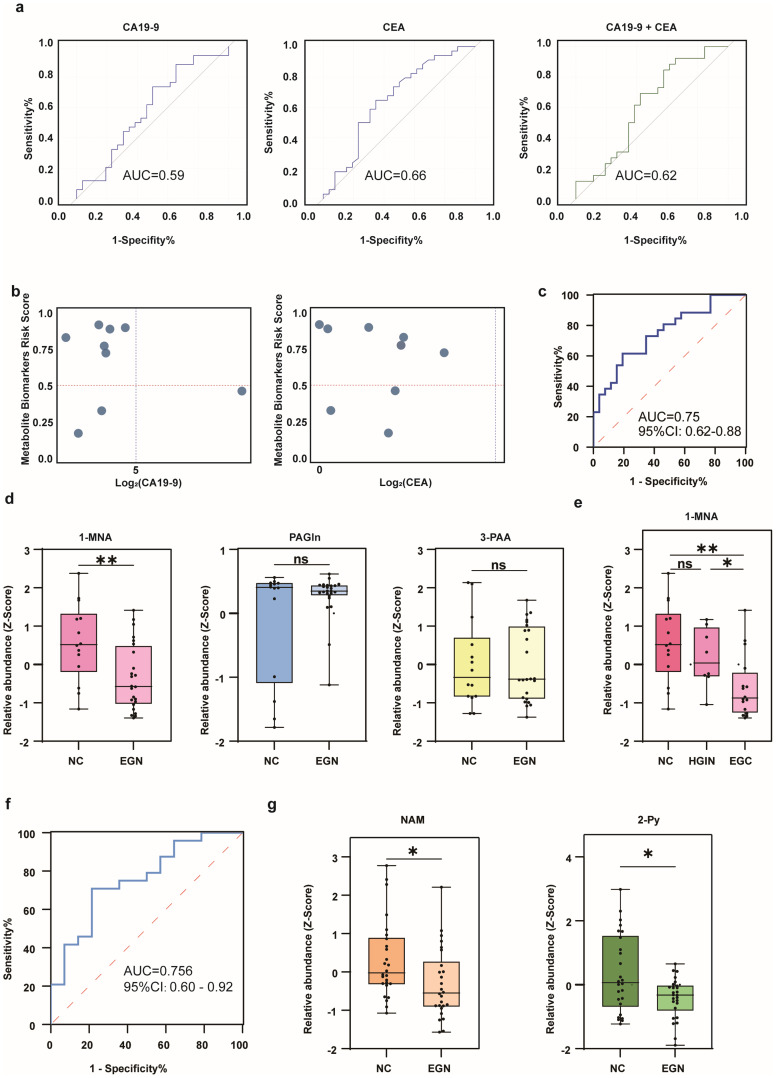
Evaluation of metabolite biomarkers for gastric cancer diagnosis. **(a)** ROC curves for CA19-9, CEA, and their combination with respective AUC values **(b)** Scatter plots of metabolite biomarker risk scores against log-transformed CA19–9 and CEA values **(c)** ROC curve of a logistic regression two-variable diagnostic model, yielding an AUC of 0.75 (95% CI: 0.62-0.88) **(d)** Box plots comparing relative abundances of 1-MNA, PAGln, and 3-PAA between NC and EGN in the external validation cohort. **(e)** Box plots comparing 1-MNA levels across NC, HGIN, and EGC **(f)** ROC curve for 1-MNA, with an AUC of 0.756 (95% CI: 0.60-0.92) **(g)** Box plots of the relative abundance of NAM and 2-Py between NC and EGN groups.

The most commonly used clinical biomarkers for diagnosing gastrointestinal tumors include CA19-9, CA125, and CEA. To evaluate the diagnostic performance of the 3-DM, serum tumor marker data (mainly CA19–9 and CEA) were collected from all participants and compared with the model’s outputs. The results showed that the AUC values for CA19–9 and CEA were 0.59 and 0.66, respectively, while their combination resulted in an AUC of 0.62, all of which were lower than the 3-DM (**Figure 6a**). To further evaluate practical diagnostic usefulness, classification performance was assessed in the internal testing set. For conventional serum tumor markers, positivity was defined according to the clinical reference thresholds used in our hospital laboratory: CA19-9 > 37 U/mL and CEA > 5 ng/mL. In the internal testing set, the 3-DM achieved an overall classification accuracy of 67% using the probability threshold of 0.5. In contrast, using the conventional clinical cutoffs, CA19–9 and CEA showed positive detection rates of 11% and 0% among EGN cases, respectively. Specifically, no HGIN or EGC case in the internal testing set had CEA above the clinical cutoff, consistent with the limited sensitivity of CEA for early-stage gastric neoplastic lesions. Therefore, the 0% value refers to the positive detection rate for EGN cases, not the overall classification accuracy (**Figure 6b**). These findings suggest that the urinary 3-DM may provide better discrimination than traditional serum tumor markers in the early detection of cancer.

### 1-MNA as an independent biomarker for EGN screening

To analyze the independent contributions of each metabolite, logistic regression with variable selection was performed on 1-MNA, 3-PAA, and PAGln. Likelihood ratio tests showed that removing either 1-MNA (*p* = 0.014) or 3-PAA (*p* = 0.012) significantly impaired model performance, while excluding PAGln had no significant effect (*p* = 0.139). Therefore, PAGln was excluded, and a two-variable model including only 1-MNA and 3-PAA was constructed (**Table 2**).

**Table 2:** Parameters of the final two-metabolite logistic regression mode (1-MNA and 3-PAA).

Variable name	Coefficient (B)	Standard Error	Wald	*p* value	OR^***^ (Exp(B)^#^)
1-MNA^*^	-0.538	0.241	4.980	0.026	0.584
3-PAA^**^	1.053	0.472	4.969	0.026	2.866

1-MNA^*^, 1-methylnicotinamide; 3-PAA^**^, 3-pyridylacetic acid; OR^***^, odds ratio; Exp(B)^#^, exponentiated regression coefficient; B, regression coefficient.

In this function, 1-MNA (variable A) was assigned a negative coefficient, indicating an inverse relationship with EGC occurrence, while 3-PAA (variable B) had a positive coefficient, indicating a direct association with EGC. The ROC curve for this two-variable model yielded an AUC of 0.75 (95% CI: 0.62-0.88) (**Figure 6c**), showing potential discriminatory power.

To evaluate the reproducibility of the candidate metabolites in an independent cohort, targeted quantification of 1-MNA, 3-PAA, and PAGln was performed on 38 urine samples, with relative abundance expressed as Z-scores. Notably, among the three metabolites included in the 3-DM, only 1-MNA indicated a statistically significant difference between NC and EGN in the independent temporal validation cohort, with levels showing a progressive decline across disease progression (NC > HGIN > EGC). 3-PAA and PAGln did not show consistent discriminatory performance across cohorts. Therefore, the cross-cohort reproducibility of the current biomarker signal appeared to be driven primarily by 1-MNA, whereas the contributions of 3-PAA and PAGln should be considered exploratory and require further validation. (**Figures 6d, e**). In the external validation set, the ROC curve for 1-MNA showed an AUC of 0.756 (95% CI: 0.60-0.92) (**Figure 6f**), indicating its potential and stability in differentiating EGN from NC. In comparison, 3-PAA and PAGln did not demonstrate consistent discriminative performance in this cohort and were not considered as independent biomarkers. Other metabolites (NAM and 2-Py) involved in the nicotinamide metabolism pathway mediated by 1-MNA also showed a decreasing trend in the EGN group (**Figure 6g**).

This study developed a lightweight diagnostic model that incorporates three urinary metabolites showing numerically higher AUROC values. To simplify testing and improve clinical applicability, independent validation was carried out, ultimately identifying 1-MNA as the most reliable and reproducible biomarker. These results highlight 1-MNA’s potential as a clinically applicable urinary biomarker for the early detection of EGN.

## Discussion

Our findings are consistent with recent progress in molecular and metabolic cancer diagnostics. Prior single-cell studies have shown that gastric carcinogenesis involves marked immune and transcriptional heterogeneity during the transition from chronic inflammation to neoplasia, supporting the need for biomarkers that can capture early biological alterations. Recent work on AI-assisted tumor microenvironment analysis further demonstrates the value of machine learning for extracting clinically informative patterns from complex molecular data (22). In gastric cancer, metabolic fingerprinting has enabled rapid, label-free histopathological assessment and prognostic prediction, highlighting the diagnostic potential of tumor-associated metabolic states (23). In addition, sustainable metabolic cancer diagnostic platforms based on dried serum spots and mass spectrometry have shown that metabolic profiling can be adapted toward rapid, affordable, and scalable cancer detection. Compared with these tissue- or serum-based strategies, our study focused on urine, a non-invasive and repeatable biospecimen, and explored whether urinary metabolomic signatures could identify clinically actionable early gastric neoplasia (24). Thus, our work complements these advances by extending metabolic diagnostic strategies toward urine-based screening of early gastric neoplastic lesions.

This study explored urinary metabolic changes in patients with EGN compared to normal controls (NCs), leading to the development of a three-metabolite diagnostic model (3-DM). Internal testing set suggested that this model had discriminative potential and showed numerically higher AUROC values than conventional serum tumor markers in this cohort. Further analysis and targeted quantification in an independent temporal validation cohort identified 1-MNA as the most reproducible single-metabolite candidate. Its levels decreased from NC to HGIN and EGC, suggesting that 1-MNA may reflect early disease-associated metabolic alterations.

Early diagnosis of GC, especially at the EGC stage, is crucial for improving patient survival (25, 26). Previous studies have explored nucleic acids, proteins, and various other serum-derived and tissue-derived molecules as biomarkers for GC. However, these candidates often face challenges such as invasiveness, poor reproducibility, and limited specificity (27–31). Considering the close association between tumor progression and metabolic activity, metabolites are promising predictive indicators for GC (29, 32). In this study, urinary metabolite profiling provided a non-invasive approach that overcomes the limitations of traditional biomarkers, allowing for detecting clinically relevant early gastric neoplastic lesions (7). Moreover, levels of 1-MNA showed a consistent decrease in different pathological categories, supporting its potential value as a candidate biomarker for further validation. Most previous studies have focused on biomarker discovery in advanced GC, while the feasibility and diagnostic needs of early-stage disease have received less attention (11, 28, 30). This study specifically targeted EGC and successfully identified diagnostic metabolites by combining urinary metabolomics with machine learning. This method showed clinical potentials by improving diagnostic sensitivity and supporting timely clinical intervention.

The diagnostic model in this study demonstrated promising performance. However, several limitations should be acknowledged. First, the sample size was relatively limited, particularly after the discovery cohort was split into training and internal testing sets and after further stratification into NC, HGIN, and EGC subgroups. This study was designed as an exploratory diagnostic study based on retrospectively retrieved archived specimens, so no formal *a priori* sample-size calculation was performed. Therefore, the current findings should be interpreted as preliminary and hypothesis-generating rather than definitive evidence of clinical diagnostic performance.

Second, although the initial untargeted metabolomics dataset was high-dimensional, we attempted to reduce overfitting by applying feature selection and building a lightweight model based on only three selected metabolites. The final machine-learning model was derived during exploratory model development rather than through prespecified cross-validation-based hyperparameter optimization. Therefore, optimism bias and model-selection uncertainty cannot be fully excluded. The internal testing set contained only 18 participants, resulting in a wide AUROC confidence interval and substantial statistical uncertainty. In addition, repeated stratified 5-fold cross-validation of the fixed three-metabolite classifier yielded a mean AUROC of 0.680, with a broad empirical percentile range of 0.440–0.960. This variability indicates that the estimated performance was sensitive to sample partitioning and that the AUROC of 0.809 obtained from the single internal testing split may partly reflect sampling variability. The diagnostic performance of the 3-DM should be interpreted cautiously. Moreover, although the 3-DM included 1-MNA, 3-PAA, and PAGln, only 1-MNA remained statistically significant in the independent temporal validation cohort. The cross-cohort robustness of the current findings therefore appears to rely primarily on 1-MNA, whereas 3-PAA and PAGln showed inconsistent performance and should be regarded as exploratory components of the discovery model. Larger prospective cohorts are required to determine whether a multi-metabolite model provides incremental value beyond 1-MNA alone. An important issue is the diagnostic specificity of 1-MNA. 1-MNA is a downstream metabolite of the niacin/nicotinamide pathway and may be influenced by dietary niacin intake, renal function, smoking status, medications, and other malignancies. In the present study, several available clinical variables potentially related to urinary metabolite levels were compared between the NC and EGN groups. Smoking status, alcohol consumption, BMI, blood glucose, lipid profiles, liver function indices, renal function indices, and uric acid were generally comparable between groups. In particular, serum creatinine and blood urea nitrogen did not differ significantly between groups, suggesting that the observed reduction in urinary 1-MNA was unlikely to be driven solely by major differences in renal function. However, there is no deeper evidence to suggest that the decline of 1-MNA is a unique metabolic change specific to early-stage gastric cancer. Detailed dietary niacin intake, over-the-counter niacin or nicotinamide supplement use, complete medication exposure, and disease-control groups with benign gastric diseases, renal dysfunction, or other malignancies were not systematically included. Therefore, the diagnostic specificity of 1-MNA for EGN remains uncertain and requires validation in larger cohorts with more comprehensive assessment of dietary, renal, medication-related, and competing disease factors.

HGIN is a high-grade non-invasive epithelial neoplastic lesion, whereas EGC is an invasive adenocarcinoma confined to the mucosa or submucosa. Therefore, combining HGIN and EGC may have introduced biological and pathological heterogeneity and may have affected both model performance and clinical interpretability. In this study, this grouping was used because both HGIN and EGC represent clinically actionable early gastric neoplastic lesions that generally require close endoscopic management or resection. Accordingly, the model should be interpreted as a preliminary tool for distinguishing non-neoplastic controls from early gastric neoplasia, rather than as a classifier that separates HGIN from EGC. Consistent with this interpretation, predicted probabilities did not clearly separate HGIN from EGC, and the subgroup analyses were exploratory because of the limited sample size. Future studies with larger cohorts should evaluate HGIN and EGC separately and develop stage-specific or multiclass models if sufficient sample size is available.

Finally, the relatively short follow-up period may have limited the scope of clinical observations. Longer follow-up is required to evaluate dynamic changes in 1-MNA during gastric cancer progression and to further examine its diagnostic and monitoring value across different clinical stages.

Metabolic pathway analysis in EGC revealed an overall downregulation of the 1-MNA pathway. Both NAM and its oxidative metabolite 2-Py were reduced along with 1-MNA, while upstream precursors nicotinamide riboside (NR) and nicotinamide mononucleotide (NMN) remained unchanged. This pattern suggests that limited NAM release, rather than a lack of precursor supply, may contribute to pathway suppression. This pattern may be related to altered NAD^+^-dependent metabolism. For example, reduced SIRT2-related NAD^+^ turnover could theoretically decrease NAM production and thereby reduce downstream 1-MNA pathway flux (33, 34). However, this mechanism remains speculative and requires direct experimental validation.

## Conclusion

This study combined urinary metabolomics and machine learning to explore a non-invasive approach for detecting early gastric neoplasia. The identified alterations in the nicotinamide pathway and their association with EGN provide preliminary insights into metabolic reprogramming during early gastric neoplastic progression. Among the candidate metabolites, 1-MNA showed reproducible changes across cohorts and may serve as a candidate urinary biomarker for EGN detection. However, its diagnostic performance and clinical applicability require further validation in larger, multicenter, prospectively designed studies.

## Data Availability

The datasets presented in this study can be found in online repositories. The names of the repository/repositories and accession number(s) can be found in the article/**Supplementary Material**.

## Supplementary material

Supplementary Table 1 Untargeted quantification data for urinary metabolites.

Supplementary Table 2 Representative misclassifications in the internal test validation set.

Supplementary Figure 1 Exploratory calibration plot of the 3-DM in the internal testing set.

Supplementary Figure 2 Distribution of fold-level AUROC estimates from repeated 5-fold cross-validation.
