# Supplementary material for: Urinary metabolomics-based machine learning for diagnosis of early gastric neoplasia: a retrospective diagnostic case-control study
Source: Front Oncol. 2026 Aug 4;16:1896373. doi: 10.3389/fonc.2026.1896373 (PMC13481301; doi:10.3389/fonc.2026.1896373)
Supplement: Supplementary file 1 [file Table2.docx]

**Table S2. Representative misclassifications in the internal test validation set**

| **Case ID** | **True label** | **Predicted label** | **Pred. prob.** | **Decision threshold** | **Error type (FP*/FN**)** | **1-MNA** | **3-PAA** | **PAGln** |
| --- | --- | --- | --- | --- | --- | --- | --- | --- |
| 7N | 0 | 1 | 0.52 | 0.50 | FP | 2.24 | 0.76 | 10.43 |
| 37G | 1 | 0 | 0.43 | 0.50 | FN | 2.10 | 1.32 | 5.36 |
| 40G | 1 | 0 | 0.42 | 0.50 | FN | 3.47 | 0.55 | 3.89 |

FP*, false positive; FN**, false negative. Pred. prob. refers to the model-assigned probability of the positive class. Values for 1-MNA, 3-PAA, PAGln are normalized intensity.
